# Efficacy of autologous stromal vascular fraction injection in the treatment of androgenic alopecia

**DOI:** 10.1007/s00403-022-02501-5

**Published:** 2022-12-16

**Authors:** Mohamed El-Khalawany, Mahmoud A. Rageh, Ibrahim Elnokrashy, Shady Mahmoud Attia Ibrahim

**Affiliations:** Department of Dermatology, Faculty of Medicine, Al-Azhar University, Cairo, Egypt

**Keywords:** Fat grafting, Stromal vascular fraction, Stem cells, Androgenic alopecia

## Abstract

Androgenic alopecia (AGA) is a common condition associated with loss of terminal hair on the scalp in a specific pattern in both males and females. Management of AGA is usually challenging as the approved therapeutic options are limited. Our aim was to evaluate the efficacy of non-enzymatic stromal vascular fraction (SVF) as a new promising treatment for AGA. From April to December 2021, this prospective study included 30 patients with AGA who were enrolled from the University Hospitals' dermatology outpatient clinics. Patients received a single session of autologous SVF injection and were then followed up for 6 months. There was an increase in hair shaft caliber from 0.037 ± 0.01 mm before treatment with SVF to 0.056 ± 0.02 mm after 6 months of treatment. Also, hair count/cm^2^ increased from 130.87 ± 14/cm^2^ to 151.93 ± 22.36/cm^2^ and terminal to vellus hair ratio increased from 77.06 ± 10.47% to 81.45 ± 11.98% at the end of the study. No significant difference was recorded between male and female groups as regard response to treatment. We concluded that SVF is an effective line of treatment for AGA with significant improvement regarding hair density, hair diameter, global photography, and patient satisfaction.

## Introduction

Androgenic alopecia (AGA) is the most prominent type of progressive hair loss in humans. Hormonal factors in addition to genetic propensity are considered the leading potential causes of this condition. Several recent advancements in molecular biology and genetics have expanded our understanding of the processes of hair loss in androgenic alopecia [1].

Medication and hair transplantation are two of the most common hair restoration methods [2, 3]. These methods are most of the time inefficient due to the high cost, possible adverse effects, unsatisfying outcomes, and the need for long-term medication use. Therefore, modern, effective, and long-lasting therapeutic strategies that work for both sexes are needed [4, 5].

Autologous fat is used for a variety of medical applications, including volume augmentation, facial contouring, and tissue rejuvenation. Apart from the lipofilling property of adipocytes, advances in fat preparation and processing have been promoted as a means of more anti-aging properties. This new era in fat use began in 2001 when Zuk et al. discussed the multipotent progenitor cells known as adipose-derived stem cells (ADSCs) [6].

Recently, the utilization of adipose tissue and ADSCs in regenerative medicine is becoming more common in all parts of medicine. Loss of hair and thickness of the scalp subcutaneous fat have been linked, as the decrease in scalp thickness and loss of its fat was found to be associated with hair loss. In addition, research on the effect of ADSCs on hair growth has shown that adipose tissue is an important element of the normal hair cycle [7, 8].

Stromal vascular fraction (SVF) contains several types of regenerative cells, including mesenchymal stem cells (MSCs), which are highly proliferative, pluripotent stem cells that have immunomodulatory, and immunosuppressive properties. Furthermore, the cells within SVF secrete several growth factors that have a variety of functions, including hair follicle activation [9].

By homing to the hair follicles and exerting different paracrine effects, SVF can have a major influence on miniaturized hair follicles [5].

New techniques for isolating SVF are identified; this can be done either enzymatically or mechanically. The enzyme-dependent method produces proteolysis of bonds between cells that enable in vitro separation of mature adipocytes from the SVF. When the process is done mechanically, fat cells are broken through mechanical emulsification and filtration, leaving the viable SVF intact [10].

It has been noted that, in producing of SVF by enzymatic method, the enzyme does not just destroy bonds but also damages stromal cells which environ the stem cell and allow interaction with adjacent cells, promoting cell preservation, proliferation, and differentiation [5]. For that reason, in addition to the higher cost and time consumption, mechanical stromal cell production is usually used [11].

The purpose of this study was to assess the efficacy of non-enzymatic SVF in treating androgenic alopecia.

## Patients and methods

### Study design and population

This prospective study included 30 patients suffering from androgenic alopecia that were recruited from the University Hospitals' outpatient clinics after the approval of the Institutional Review Board of the Faculty of Medicine. The study protocol conformed to the ethical guidelines of the 1975 Declaration of Helsinki. All patients were treated with a single session of autologous SVF injection and were then followed up for a period of 6 months.

All participants were fully informed about the nature of this research, the anticipated results, and any potential complications before providing written informed consent.

### Clinical evaluation

All patients included in the study were subjected to complete history taking, thorough general and dermatological examinations, and routine laboratory investigations.

Patients younger than 18 years; patients who received minoxidil or any other oral, topical medications (including herbal medications) or injection procedures for the treatment of hair loss within 6 months prior to the study, or finasteride or dutasteride within 12 months of the study; patients suffering from dermatological condition or a significant scarring in the treatment area; patients with hematological disorders, severe chronic illnesses, malignancy, allergic or autoimmune diseases; pregnant, and lactating women were all excluded from the study.

### SVF preparation

Coleman's technique was used for fat grafting [12]. First, Klein's [13] solution of tumescent anesthesia was administered using (500 ml of 0.9% sodium chloride, 25 ml of 2% lidocaine, and 0.5 ml of epinephrine). After that, fat tissue was extracted with a Sorensen harvester cannula (Tulip Medical Inc.) attached to a 20-ml Luer-Lock syringe. Suction was achieved by applying constant negative pressure.

After harvesting 80 ml of fat, the process of condensation started by centrifugation of lipoaspirate (using 80–1 Electric Centrifuge, Jiangsu Jinyi Instrument Technology Company Limited) at 500 g-force for 2 min, and this was enough to isolate the tumescent fluid and the blood elements in the lowest layer which was then discarded.

After centrifugation, the lipoaspirate was mechanically emulsified using Tulip NanoTransfer kit through 2.4, 1.4, and 1.2 mm Luer-to-Luer connectors, respectively, with 30 passes through each connector using minimal pressure force in order to achieve successful mechanical micronization of fat.

The micronized fat underwent another centrifugation at 1200 g-force for 3 min to eliminate the oil. Then, the remaining emulsified fat was filtered by passing onetime through a double layered filter of 400 and 600 μm. For the last time, the product was centrifuged at 1200 g-force for 6 min, as a result of this process, 3 layers were obtained with SVF pellet in the bottom. After isolation of SVF, the scalp was injected under strict sterile conditions with 0.1 ml/cm^2^ of SVF intradermally using 30 G syringes.

Patients were assessed one week after the procedure for detection any side effects like pain, edema, and/or bruises following the procedure of fat harvesting. Full evaluation was done at the 6-month follow-up visit.

### Response to treatment

All patients were assessed by digital trichoscopic examination of the scalp (using Dlite STR CA_USA) before treatment and at the 6-month follow-up visit to evaluate both hair density and hair shaft thickness, and terminal to vellus hair ratio. Digital images were taken at reference points using a headband and a tapeline as proposed by Lee et al. [14].

Also, global photography was assessed by 2 independent non-treating blinded dermatologists who were asked to subjectively compare digital photographs which were taken by the same photographer at the same distance each time using Canon digital camera (EOS 800EF-S 18-55 mm F4-5.60 IS STM lens-24.2MP DSLR) at 6 months after treatment with baseline photographs regarding hair condition as follows: Great worsening =  – 3, Moderate worsening =  – 2, Slight worsening =  – 1, Stabilization = 0, Slight improvement = 1, Moderate improvement = 2, Great improvement = 3.

### Patient satisfaction

All patients were asked to evaluate their own level of satisfaction by giving themselves a score from 0 to 3 points (0 = slight improvement, 1 = moderate improvement, 2 = significant improvement, and 3 = marked improvement).

### Assessment of pain

Patients were asked to score the level of pain they experienced during the procedure of fat harvesting and SVF injection at a range of 0–3 points (0 = no pain, 1 = mild pain, 2 = moderate pain, and 3 = severe pain).

### Statistical analysis

Data were analyzed using Statistical Program for Social Science (SPSS) version 25. Appropriate analysis was performed for every variable based on the type of data collected.

## Results

This study was done on 30 patients having androgenic alopecia, 14 (46.7%) males and 16 (53.3%) females, all treated with a single injection of non-enzymatic SVF. The mean age of all studied patients was 30.1 ± 6.3 years with a minimum age of 21 years and maximum age of 45 years. Nineteen (63.3%) patients had a family history of AGA, whereas the remaining 11 patients (36.7%) were experiencing active hair loss with no family history of AGA. The abdomen was the chosen site for fat harvesting in all patients.

Ludwig scale assessment in female patients revealed, 7 patients (43.8%) in grade I, 5 patients (31.2%) in grade II, and 4 patients (25%) in grade III. On the other hand, the Hamilton-Norwood scale assessment in male patients showed that there were 3 patients (21.4%) in grade I, 4 patients (28.6%) in grade II, 2 patients (14.3%) in grade III, 1 patient (7.1%) in grade IV, 3 patients (21.4%) in grade V, and 1 patient (7.1%) in grade VI (Table 1).

**Table 1:** Description of demographic data in all studied patients

		Studied patients (*N* = 30)	
Sex	Male	14	46.7%
	Female	16	53.3%
Age (years)	Mean ± SD	30.1 ± 6.3	
	Min—Max	21 – 45	
Grade of AGA in females according to Ludwig scale	Grade I	7	43.8%
	Grade II	5	31.2%
	Grade III	4	25%
Grade of AGA in males according to Hamilton-Norwood scale	Grade I	3	21.4%
	Grade II	4	28.6%
	Grade III	2	14.3%
	Grade IV	1	7.1%
	Grade V	3	21.4%
	Grade VI	1	7.1%

*AGA* androgenic alopecia

**Fig. 1:**
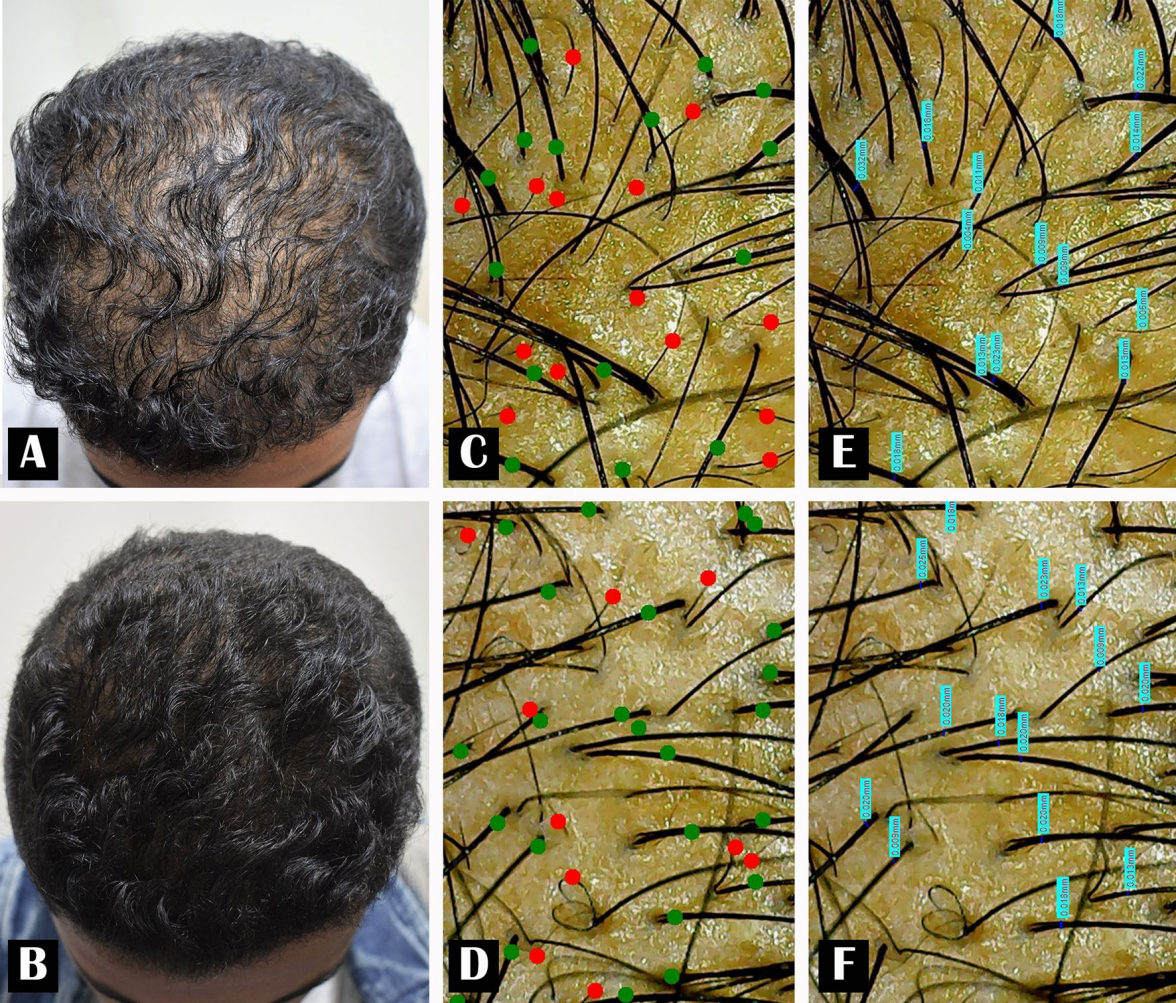
Male patient 24 years old. **A** Before treatment. **B** Six months after SVF injection. **C**, **E** Digital trichoscopic images before treatment. **D**, **F** Improvement in hair density and hair thickness after 6 months of SVF treatment, (green dots = terminal hair, red dots = vellus hair)

**Fig. 2:**
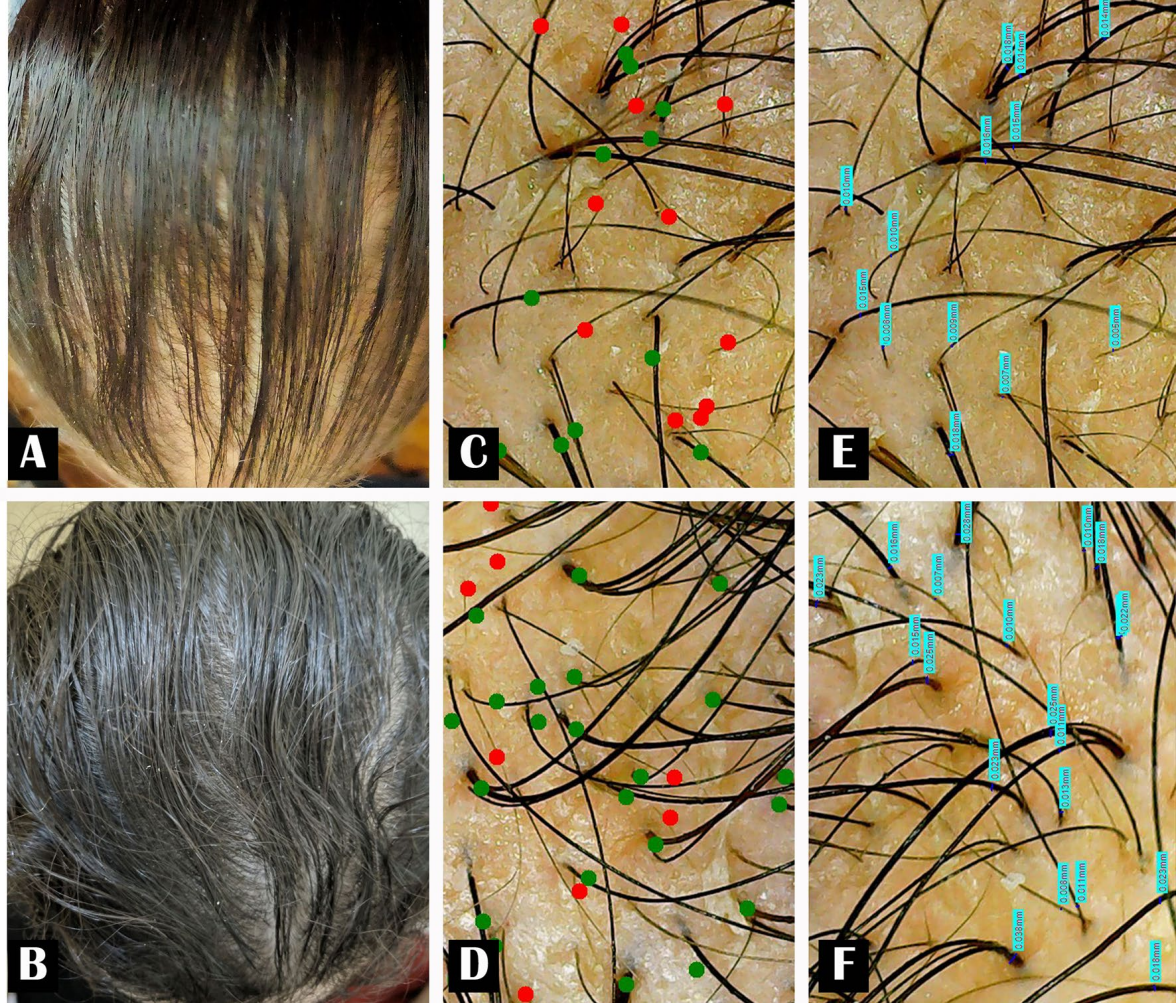
Female patient 32 years old. **A** Before treatment. **B** Six months after SVF injection. **C**, **E** Digital trichoscopic images before treatment. **D**, **F** Improvement in hair density and hair thickness after 6 months of SVF treatment

As regards digital trichoscopic examination, the hair shaft caliber showed a high statistically significant increase from 0.037 ± 0.01 mm before treatment to 0.056 ± 0.02 mm after 6 months of SVF injection with a 51.35% improvement. Also, hair count/cm^2^ showed a high statistically significant increase from 130.87 ± 14/cm^2^ before the study to 151.93 ± 22.36/cm^2^ at the 6-month follow-up visit with a 16.09% improvement. Moreover, the terminal to vellus hair ratio increased significantly from 77.06 ± 10.47% before the study to 81.45 ± 11.98% at the end of the study with a 5.7% improvement (Figs. 1 and 2) (Table 2).

**Table 2:** Comparisons of digital trichoscopic findings before and after treatment in all studied patients

		Before (*N* = 30)	After (*N* = 30)	Statistical test	*p*-value
Average shaft caliber (mm)	Mean	0.037	0.056	T = 4.6	< 0.001 HS
	± SD	0.01	0.02		
Hair count (/mm)	Mean	130.87	151.93	T = 4.3	< 0.001 HS
	± SD	14.00	22.36		
Terminal/ vellus hair ratio	Mean	77.06	81.45	MW = 272	0.008 S
	± SD	10.47	11.98		

*T*, independent sample *t*-test, *MW* Mann–Whitney *U* test, *HS* (pink colored cells), *p*-value < 0.001 is considered highly significant, S (green colored cells), *p*-value < 0.05 is considered significant

Global photography assessment of the studied patients by 2 independent dermatologists revealed that 5 patients (16.7%) were stable, 4 patients (13.3%) showed slight improvement, 15 patients (50%) showed moderate improvement, and 6 patients (20%) showed great improvement.

As regards patient satisfaction, 3 patients (10%) reported slight improvement, 5 patients (16.7%) reported moderate improvement, 16 patients (53.3%) reported significant improvement, and 6 patients (20%) reported marked improvement.

Regarding pain assessment during and after the procedure, 21 patients (70%) reported mild pain, 9 (30%) reported moderate pain, and none reported severe pain. No other side effects were observed in any of the enrolled patients.

No remarkable difference was recorded between male and female patients as regard response to therapy (Table 3).

**Table 3:** Comparisons between males and females regarding digital trichoscopic findings, global photography, and patient satisfaction

				Males (*N* = 14)	Females (*N* = 16)	Statistical test	*p*-value
Before	Average shaft caliber (mm)	Mean	0.034	0.041	T = 1.92	0.065 NS	
		± SD	0.01	0.01			
	Hair count (/mm)	Mean	125.4	135.6	T = 2.1	0.044 S	
		± SD	16.7	9.2			
	Terminal/vellus hair ratio	Median	81	81	MW = 112	1.0 NS	
		IQR	61–85	76–84			
After	Average shaft caliber (mm)	Mean	0.051	0.061	T = 1.5	0.133 NS	
		± SD	0.02	0.02			
	Hair count (/mm)	Median	148	161	MW = 69.5	0.077 NS	
		IQR	129– 161	154–165			
	Terminal/vellus hair ratio	Median	83	87	MW = 87.5	0.313 NS	
		IQR	64–89	83–90			
Global photography	Stable	3	21.4%	2	12.5%	X^2^ = 2.74	0.433 NS
	Slight improvement	3	21.4%	1	6.3%		
	Moderate improvement	5	35.7%	10	62.5%		
	Great improvement	3	21.4%	3	18.8%		
Patient satisfaction	Slight improvement	2	14.3%	1	6.3%	X^2^ = 1.4	0.704 NS
	Moderate improvement	3	21.4%	2	12.5%		
	Significant improvement	6	42.9%	10	62.5%		
	Marked improvement	3	21.4%	3	18.8%		

*IQR* interquartile range, *T* independent sample *t*-test, *MW* Mann–Whitney *U* test, χ2 chi-square test, *NS* *p*-value > 0.05 is considered non-significant; S (green colored cells), *p*-value < 0.05 is considered significant

## Discussion

Because of the high concentration of stem cells and relative ease of access, adipose tissue has attracted a lot of attention among the various sources of MSCs. As a result, fat grafting is now widely used in the field of plastic surgery and is gaining popularity in the field of regenerative medicine, particularly in dermatology [15].

MSCs are important for maintaining the stem cell niche, which includes hair follicular stem cells, as well as prolonging the anagen phase [16]. Most research on adipose tissue derivatives in alopecia treatment has relied on the paracrine function of ADSCs and SVF cells. In vitro, ex vivo, and in vivo, the ADSC-secretome enhances hair growth [17].

Furthermore, ADSC-derived proteins help to preserve dermal papilla cells from androgens and reactive oxygen species-induced cytotoxicity [8]. ADSCs have an antiandrogen effect via the isoenzyme aldo–keto reductase 1C2 (AKR1C2), which inactivates androgens by converting potent DHT into weak 3-alpha diol through 3-alpha reductase activity. Adipose tissue, when injected into a hair loss area, could have an antiandrogen impact without causing systemic effects [18].

The production of bioactive factors involved in the hair growth cycle and hair differentiation, such as IGF, VEGF, HGF, PDGF, and Wnt pathway regulating factors, explains the action of ADSCs [8, 9]. Wnt3a promotes hair follicle growth by activating the Wnt/β-catenin signaling pathway, which is vital for anagen initiation [19, 20].

ADSC therapy has recently been proposed as a way to improve aging-related alopecia while maintaining mitochondrial quality control through mitophagy regulation [8].

SVF is a heterogeneous group of stem/stromal cell lines derived from the adipose tissue complex's perivascular and extracellular matrix. Because of its lack of immunogenic reactions, ease of extraction, multipotential characteristics, availability of separating it into different cell lines, and substantial angiogenesis capacity, the SVF is best suited for use in regenerative surgery [7].

The results of our study showed that SVF injection led to significant improvement in hair shaft caliber, hair count/cm^2^, and terminal to vellus hair ratio. The patients noticed a reduction in hair loss and an increase in hair density. This could be due to a cellular environment in which hair cells have a rich microcirculation. SVF and ADSCs have been found to stimulate angiogenesis [21, 22].

On reviewing the literature, we found few studies that evaluate SVF efficacy in the treatment of androgenic alopecia.

In a study by Kim et al. [23], they treated 9 patients with a single injection of SVF in the vertex of the scalp and found a significant increase in hair diameter and hair count after 6 months of therapy. This comes in agreement with our study.

Another study was done by Perez-Meza et al. [24], they brought 9 patients with different grades of AGA and tried to use SVF-enhanced adipose tissue injection as a treatment. Only 6 patients had completed the study. Also, an inadequate amount of aspirated fat was harvested in 1 patient, so this patient received fat alone without SVF. Their results showed a mean increase of 31 hairs/cm^2^ of scalp was found in patients treated with fat plus SVF, while the one participant who had fat injection alone recorded a mean increase of 14 hairs/cm^2^. They concluded that while fat alone may be an efficient approach for the treatment of AGA, SVF addition may improve the outcome of therapy.

SVF efficacy in the treatment of AGA was also studied by Ozturk et al. [25]. There was an improvement in terms of hair diameter and density in 20 patients after 3 months following SVF injection.

Other studies showed that SVF can be used as a synergistic tool with other treating modalities of AGA such as platelet-rich plasma (PRP).

Butt et al. [5] evaluated SVF in 11 AGA patients. The patients were split into two groups: PRP and SVF-PRP. Patients were injected twice, four weeks apart, and the results were evaluated 6 months later. The PRP group witnessed a 21.5% increase in hair density, while in the SVF-PRP group there was a 51.6% increase. Both groups experienced a decrease in pull test, which was more significant in the SVF-PRP group. The physician and patient assessment scores in the SVF-PRP group also improved significantly.

Similarly, Stevens et al. [21] published a study that investigated the effects of SVF in combination with PRP in 10 male AGA patients. At 6 and 12 weeks after treatment, there was a significant increase in hair density. However, because there was no control group in this study, it's difficult to state that SVF is more effective than PRP.

The better overall effect of autologous SVF on hair loss can be a promising therapeutic model for both men and women. Furthermore, when combined with existing biological treatment methods like follicular stem cell therapy or PRP, the improvement of hair loss employing SVF is thought to have a superior expected effect on AGA [23].

Treatment of alopecia often necessitates multiple consecutive or simultaneous therapies, and it is notable that, in some studies, SVF improved hair loss without conjunctive therapy and after a single treatment. If long-term beneficial effects are ensured, SVF is anticipated to be a reliable strategy to cure AGA in the future, compared to conventional treatment methods, since it hinders the underlying causes of AGA.

## Conclusion

This study shows that SVF offers a huge potential for hair regeneration. There were no negative side effects reported by any of the patients. The procedure appears to be safe and well-tolerated, with a positive response. The study had some limitations, such as a small number of sessions and a short follow-up period. Despite these drawbacks, SVF enrichment may prove to be a promising alternative treatment for both men and women suffering from AGA. However, more research is needed, with a larger number of sessions and longer follow-up duration.

## Data Availability

The data that support the findings of this study are available from the corresponding author upon reasonable request.
